# Use of seaweed *Ulva lactuca* for water bioremediation and as feed additive for white shrimp *Litopenaeus vannamei*

**DOI:** 10.7717/peerj.4459

**Published:** 2018-03-05

**Authors:** Regina Elizondo-González, Eduardo Quiroz-Guzmán, Cristina Escobedo-Fregoso, Paola Magallón-Servín, Alberto Peña-Rodríguez

**Affiliations:** CONACYT-Centro de Investigaciones Biológicas del Noroeste, La Paz, Baja California Sur, México

**Keywords:** Feed additive, Seaweed, Shrimp, Water quality

## Abstract

Two experimental feeding trials were conducted during four weeks to evaluate the use of *Ulva lactuca* in shrimp culture: (1) for wastewater bioremediation, and (2) using different inclusion levels of *U. lactuca* meal in shrimp feed. In feeding trial 1, shrimp reared under seaweed *U. lactuca* water exchange in a re-circulation system (SWE) resulted in similar growth and feed utilization as shrimp reared with clean water exchange (CWE). Shrimp under no water exchange (NWE) resulted in significant lower growth and higher feed conversion rate (FCR) compared to the other treatments (*p* < 0.05). Nitrogen compounds and phosphate in water from SWE and CWE treatments did not present significant differences during the experimental trial (*p* > 0.05). In feeding trial 2, *U. lactuca* biomass produced by wastewater bioremediation in SWE treatment were dried and ground to formulate diets containing 0, 1, 2, and 3% *U. lactuca* meal (0UL, 1UL, 2UL, and 3UL). Shrimp fed the 3 UL diet resulted in a significant (*p* < 0.05) improvement of growth and FCR, and enhanced whole shrimp lipid and carotenoid content by 30 and 60%, respectively, compared to control diet. Seaweed *U. lactuca* is suggested as a desirable species for wastewater bioremediation in integrated aquaculture systems, and its meal as a good feed additive for farmed shrimp.

## Introduction

In the last decades, aquaculture has been one of the fastest growing industries of food production. By 2015, farmed shrimp represented an estimate global production of 4.8 million metric tons, with a value of US$24.96 billion (FAO, 2017). Some of the challenges for this growing activity are the reduction of coastal water pollution impact (Herbeck et al., 2013) and the search for non-conventional ingredients to produce high quality feeds (Little, Newton & Beveridge, 2016). In the case of seaweeds produced by aquaculture, it has been estimated by 2015 a worldwide production of 28.5 million metric tons valued in US$4.46 billion (FAO, 2017). For *Ulva lactuca* seaweed, research interest has increased due to their rapid vegetative growth in the presence of high nutrient levels and has been adapted to culture in tanks (DeBusk, Blakeslee & Ryther, 1986), with various industrial applications including human and animal nutrition (Ortiz et al., 2006; Yaich et al., 2011).

The use of seaweeds in integrated multi-trophic aquaculture (IMTA) has been proposed as an alternative for environmental-sustainable expansion of aquaculture, serving as primary food source and also for water bioremediation due to their high capability of removing inorganic nutrients from wastewater (Neori et al., 2004; Neori, 2008; Fleurence et al., 2012). Benefits of integrated aquaculture of shrimp and green seaweeds has been documented for *Ulva clathrata* that showed high efficiency in removing the inorganic nutrients from water effluents (Copertino, Tormena & Seeliger, 2009), in addition as an improvement of feed utilization in white shrimp *Litopenaeus vannamei* (Cruz-Suárez et al., 2010) and in brown shrimp *Farfantepenaeus californiensis* (Peña Rodríguez et al., 2016; Peña Rodríguez et al., 2017). *U. lactuca* improved water quality when cultured with western king prawn *Penaeus latisulcatus* (Van Khoi & Fotedar, 2011) and with *L. vannamei* (Brito et al., 2014); *Caulerpa sertularioides* presence resulted in *F. californiensis* growth enhancement (Portillo-Clark et al., 2012).

Seaweeds are an excellent source of protein, carotenoids, minerals, polysaccharides, and vitamins making their utilization as feed additives attractive (Kumar et al., 2011; Peña Rodríguez et al., 2011; Syad, Shunmugiah & Kasi, 2013). Some seaweeds have been suggested as a partial feed substitute (Marinho-Soriano et al., 2007) for shrimp diet, and considered a good source of protein (Da Silva & Barbosa, 2009), which represents the most expensive fraction of feed cost. In some cases, shrimp composition is modified when fed seaweeds, these changes may include lipid content and carotenoids (Cruz-Suárez et al., 2010; Subhra Bikash, 2015), or total cholesterol (Casas-Valdez et al., 2006). The optimal level of inclusion of seaweed meal in shrimp feed varies among seaweed species, but, in most cases, studies have found benefits when included not higher than 5% (Cruz-Suárez et al., 2009; Rodríguez-González et al., 2014; Cárdenas et al., 2015; Yu et al., 2016; Schleder et al., 2017).

Despite some studies explore the use seaweeds for water bioremediation, there is no reports of the use of *U. lactuca* produced under these conditions as a feed ingredient for shrimp. The aim of the present work was to evaluate shrimp growth and water quality of an integrated culture system with *U. lactuca* and, on the other side, the effect of *U. lactuca* meal as feed additive at different inclusion levels on shrimp performance, lipid and carotenoid content.

## Materials and Methods

### Collection and maintenance of seaweed

Seaweed *U. lactuca* was collected from the La Paz bay in Baja California Sur, Mexico (Collection permit Conapesca #PRMN/DGOPA-019/2015). The *U. lactuca* was washed with sterilized marine water to remove epiphytes, then placed in laboratory conditions, in 5-L marine water tanks, at 25 °C, with a photoperiod of 12 h:12 h light:dark with fluorescent light tubes of 75 W, and using Provasoli medium at a constant concentration of 0.5 ppm of nitrogen in water. The *U. lactuca* was kept under laboratory conditions during two weeks prior to the feeding trial.

### Feeding trials

For the first experimental trial, three different treatments during 28 days were evaluated: clean water exchange (CWE), seaweed *U. lactuca* water exchange (SWE), and no water exchange (NWE). The CWE treatment consisted in 50% daily exchange of clean water using marine water pumped from an open water intake from La Paz bay, filtered up to 1-µm mesh and sterilized by UV light. For the SWE treatment, one tank of 50 L was placed with 50 g of *U. lactuca* next to a shrimp tank, making a 50% daily water exchange in a re-circulation system (using a transitory tank to make the exchange) between shrimp and *U. lactuca* tanks, without new clean water input. Each *U. lactuca* tank was provided with artificial light (cool-white fluorescent lamps 70 W; Osram) with photoperiod of 12h:12 h light:dark. The *U. lactuca* was partially harvested every week to maintain 50 g in each tank. The harvested *U. lactuca* was washed with distilled water, dried in a forced-air oven at 50 °C for 4 h, and stored at 4 °C until use for feeding trial 2. In the case of NWE treatment, only 5% of clean water was recovered in each tank per week due to evaporation. All treatments were evaluated in triplicate, and each replicate consisted of a 50-L fiberglass tank provided with aeration and temperature control containing 10 *L. vannamei* shrimp (initial weight 0.30 ± 0.05 g) obtained from a commercial hatchery (Acuacultura Mahr, S.A. de C.V.) and previously acclimated to laboratory conditions (28 °C and 35‰  salinity). Shrimp from all treatments were fed with a control feed of 34% crude protein and 8% lipids (see Table 1, treatment 0UL), with an initial rate of 10% biomass divided in two rations distributed at 9:00 and 15:00 h. After second day, the feeding rate was *ad libitum* by adjusting each tank according to consumed feed. The feed was manufactured in the Aquaculture nutrition laboratory at CIBNOR. All dry ingredients (≥250 µm) were mixed first, then oil-based ingredients and water were added and mixed again to obtain a homogenous mixture, and passed through a 2-mm die in a meat grinder. The pellets were dried in a forced-air oven at 45 °C for 12 h, and stored at 4 °C until feeding time.

**Table 1:** Ingredients, proximate composition, and gross energy of experimental diets and *Ulva lactuca* meal.

	0UL	1UL	2UL	3UL	*U. lactuca*
Ingredients					
Fish meal^a^	240	240	240	240	
Soybean meal^b^	230	230	230	230	
Wheat meal^c^	401	391	381	371	
Soy lecithin^d^	41	41	41	41	
Corn gluten^e^	30	30	30	30	
Fish oil^a^	24	24	24	24	
Vitamin premix^f^	18	18	18	18	
Grenetin^g^	10	10	10	10	
Mineral premix^h^	5	5	5	5	
Vitamin C^i^	1	1	1	1	
*Ulva lactuca* meal	0	10	20	30	
Proximate composition (g 100 g^−1^ dry matter)					
Moisture	8.3 ± 0.1	8.2 ± 0.1	8.7 ± 0.2	8.5 ± 0.1	8.7 ± 0.1
Protein	33.9 ± 0.24	33.9 ± 0.06	33.7 ± 0.10	33.5 ± 0.08	15.5 ± 0.1
Lipids	7.9 ± 0.08	7.9 ± 0.03	7.9 ± 0.06	7.9 ± 0.12	0.3 ± 0.01
Crude Fiber	0.87 ± 0.01	0.86 ± 0.06	0.86 ± 0.03	0.87 ± 0.06	3.3 ± 0.1
Ash	6.6 ± 0.03	6.9 ± 0.03	7.3 ± 0.01	7.7 ± 0.03	36.5 ± 0.1
NFE	50.6	50.3	50.2	50.0	44.5
Gross energy (MJ kg^−1^)	18.09 ± 0.47	17.93 ± 0.22	17.77 ± 0.35	17.60 ± 0.28	9.46 ± 0.14

**Notes.**

a Proteinas Marinas y Agropecuarias SA de CV, Jalisco, MX.

b Promotora Industrial Acuasistemas SA de CV (PIASA), Baja California Sur, MX.

c Molino San Cristobal, Sonora, MX.

d Suministros AZ, Baja California Sur, MX.

e Agro Insumos Basicos, SA de CV, MX.

f Vitamins: Vit. A, (20,000 UI/g) 90 mg/kg; Vit. B1, 9 mg/kg; Vit. B2, 54 mg/kg; Vit. B5, 90 mg/kg; Vit. B6, 18 mg/kg; Vit. B12, 0.04 mg/kg; Vit. K3, 36 mg/kg; Vit. D3, (850,000 UI/g) 144 mg/kg; Vit. H, 1 mg/kg; folic acid, 3.24 mg/kg; Inositol, 90 mg/kg. Sigma aldrich, Missouri, US.

g Knox, Estado de Mexico, MX.

h Minerals: CoCl_2_, 20 mg/kg; H_2_MnO_5_S, 3.3 g/kg; H_14_O_11_SZn, 66 g/kg; CuH_10_O_9_S, 1.3 g/kg; FeSO_4_, 20 g/kg; Na_2_SeO_3_, 50 mg/kg; KI, 330 mg/kg. Sigma Aldrich, Missouri, US.

i Rovimix Stay C 35%, DSM, Heerlen, NL.

Values of proximate composition are given as mean ±SD of triplicate determinations.

During the experimental period, water temperature, pH and oxygen were monitored daily with a multiparameter YSI 556 (YSI Incorporated, Yellow Springs, OH, USA). The total ammonia, nitrites, nitrates, and phosphate were measured every four days by spectrophotometric methods according to the manufacturer’s specification (LYSA, Mexico). At the end of the experimental period, shrimp performance was measured in terms of final weight, weight gain, specific growth rate (SGR), feed conversion ratio (FCR), feed consumption, and survival.

For the second experiment, a 28 day feeding trial was performed to evaluate the *U. lactuca* meal produced by water bioremediation. Based on the control diet of experiment 1, three more diets were produced including 1, 2, and 3% levels of the *U. lactuca* meal (in substitution of the same proportion of wheat meal) (see Table 1). All experimental *U.  lactuca* feeds were produced as described previously as in the control feed. Each treatment was evaluated in triplicate as described in experiment 1, using shrimps with an initial average weight of 0.59 ± 0.09 g. Feeding strategy was conducted as in the previous trial. At the end of the experimental period, five complete shrimps and five shrimps separated in cephalothorax (head) and tail from each treatment were lyophilized for total lipid and carotenoid analysis. Total lipid content was performed according to Barnes & Blackstock (1973) by using phosphosulphovanillin method and measured by spectrophotometry (Multiskan spectrum; Thermo Fisher, Vantaa, Finland) at 540 nm. Total carotenoid content was analyzed according to Palacios et al. (1999), employing acetone:methanol (2:1) for extraction and measured by spectrophotometry at 495 nm.

Water quality parameters were measured as described previously. Proximate analysis of all experiment feeds and *U. lactuca* was conducted according to AOAC (2005) methods, nitrogen free extract (NFE) was calculated through difference, and gross energy was measured with an adiabatic calorimeter. Total carotenoids from *U. lactuca* meal was analyzed as described for shrimp samples. The proximate composition of experimental feeds and *U. lactuca* meal are presented in Table 1.

### Data analysis

Data were analyzed for normality and homoscedasticity through Shapiro–Wilk and Levene’s test, respectively. Results were reported as means ± standard deviation (SD) and group means were compared using one-way analysis of variance (ANOVA) followed, if applicable, of a Tukey’s multiple comparison test (95% confidence). All data were analyzed with the SPSS Statistics 17.0 software.

## Results

**Figure 1:**
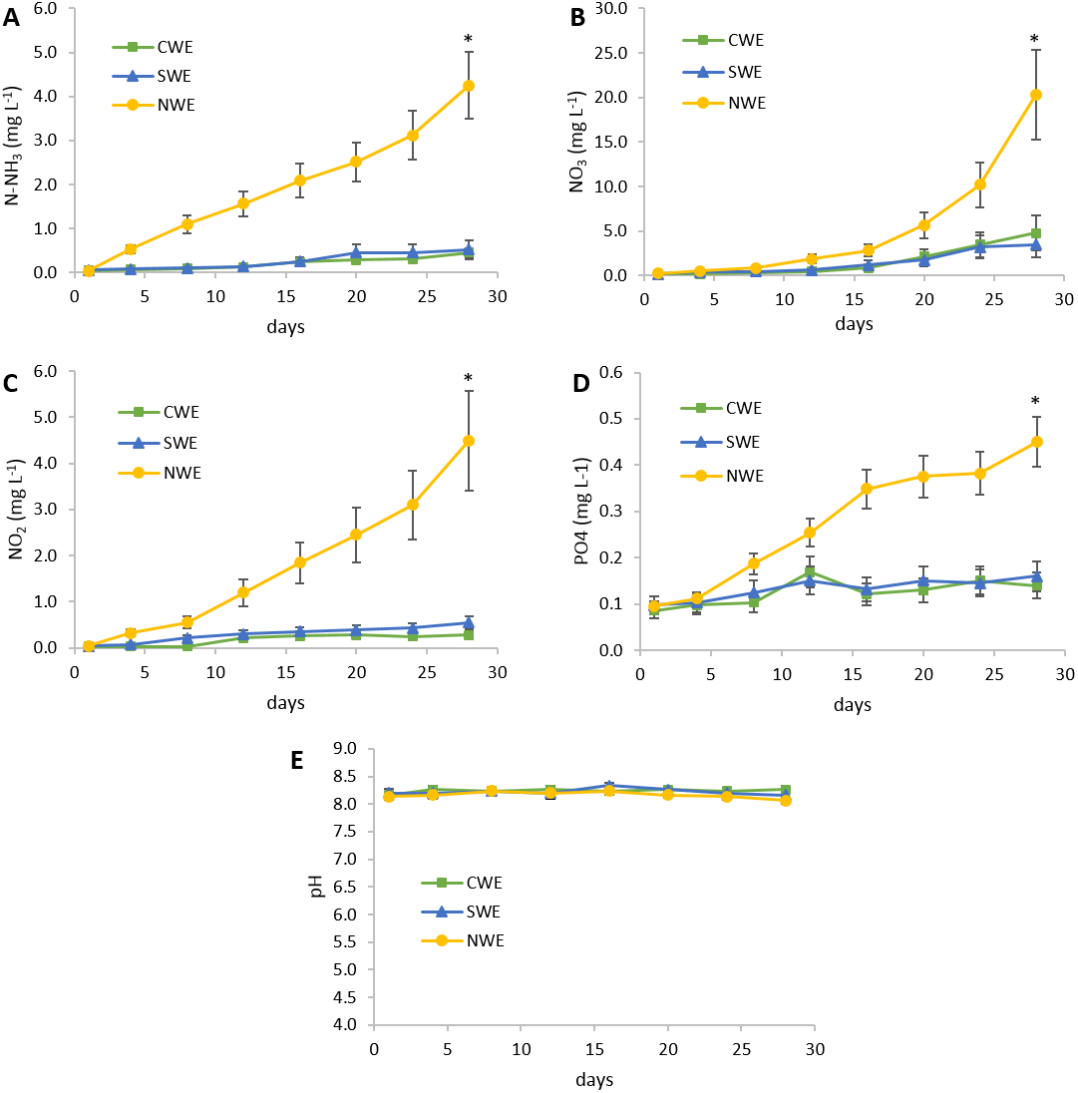
Water quality parameters during experiment 1. CWE, 50% daily clean water exchange; SWE, 50% daily exchange with water bioremediation by *Ulva lactuca*; NWE, no water exchange. (A) Water variations of total ammonia nitrogen (N-NH_3_); (B) Water variations of nitrate (NO_3_); (C) Water variations of nitrite (NO_2_); (D) Water variations of phosphate (PO_4_); (E) Water variations of pH. Values are given as mean ± SD of triplicate determinations, (∗) are significantly different (*p* < 0.05).

At the end of feeding trial 1, shrimp under daily clean water exchange (CWE) and daily seaweed *U. lactuca* water exchange (SWE) treatments resulted in significant higher (*p* < 0.05) final weight, weight gain, and SGR compared to shrimp with no water exchange (NWE) (Table 2). Feed consumption was similar among the treatments, nevertheless FCR observed in NWE treatment was significantly higher than the rest of the treatments (*p* < 0.05). Shrimp under NWE treatment showed a lower percentage of survival but not significantly different compared to the rest of treatments. At the end of the experimental period, water quality parameters were significantly different among treatments (Fig. 1). The NWE treatment resulted in significant increment (*p* < 0.05) of total ammonia nitrogen, nitrites, nitrates, and phosphate compared to treatments with water exchange, whereas the pH showed no significant differences among treatments. Removal of inorganic compounds in *U. lactuca* treated water was higher than 80% for nitrogenous compounds and 64% for phosphate compared to the treatment without water exchange. Total harvest of fresh *U. lactuca*, under the experimental conditions, was 225 ± 25 g per tank, with a specific growth rate of 5.37 ± 0.41 (% day^−1^). After drying, *U. lactuca* meal resulted in a 15.5% of crude protein and 36.5% ash (Table 1), which was used to prepare experimental feeds for feeding trial 2.

**Table 2:** Growth, feed utilization and survival after 4-week experimental trial with *L. vannamei* reared under regular water exchange (CWE), water recirculation with *U. lactuca* (SWE) and no water exchange (NWE).

	CWE	SWE	NWE	*P* value
Final weight (g)	2.15 ± 0.06^b^	2.08 ± 0.04^b^	1.82 ± 0.05^a^	0.000
Weight gain (%)	613 ± 19^b^	593 ± 12^b^	503 ± 16^a^	0.000
SGR (% day^−1^)	7.02 ± 0.10^b^	6.91 ± 0.06^b^	6.42 ± 0.10^a^	0.000
FC (g)	2.20 ± 0.08	2.11 ± 0.02	2.14 ± 0.05	0.214
FCR	1.19 ± 0.01^a^	1.18 ± 0.02^a^	1.41 ± 0.06^b^	0.000
Survival (%)	90 ± 10	96 ± 6	83 ± 6	0.171

**Notes.**

Values are given as mean ±SD of triplicate determinations. Means with different superscripts in same row are significantly different (*p* < 0.05).

Weight gain (%) (final weight-initial weight)/ initial weight × 100 SGR (% day^−1^) 100 (ln(average final weight)-ln(average initial weight))/number of days FC (g) pelleted feed consumed per shrimp FCR pelleted feed consumed (g)/wet weight gain (g) Survival (%) final number of shrimp/ initial number of shrimp × 100

**Figure 2:**
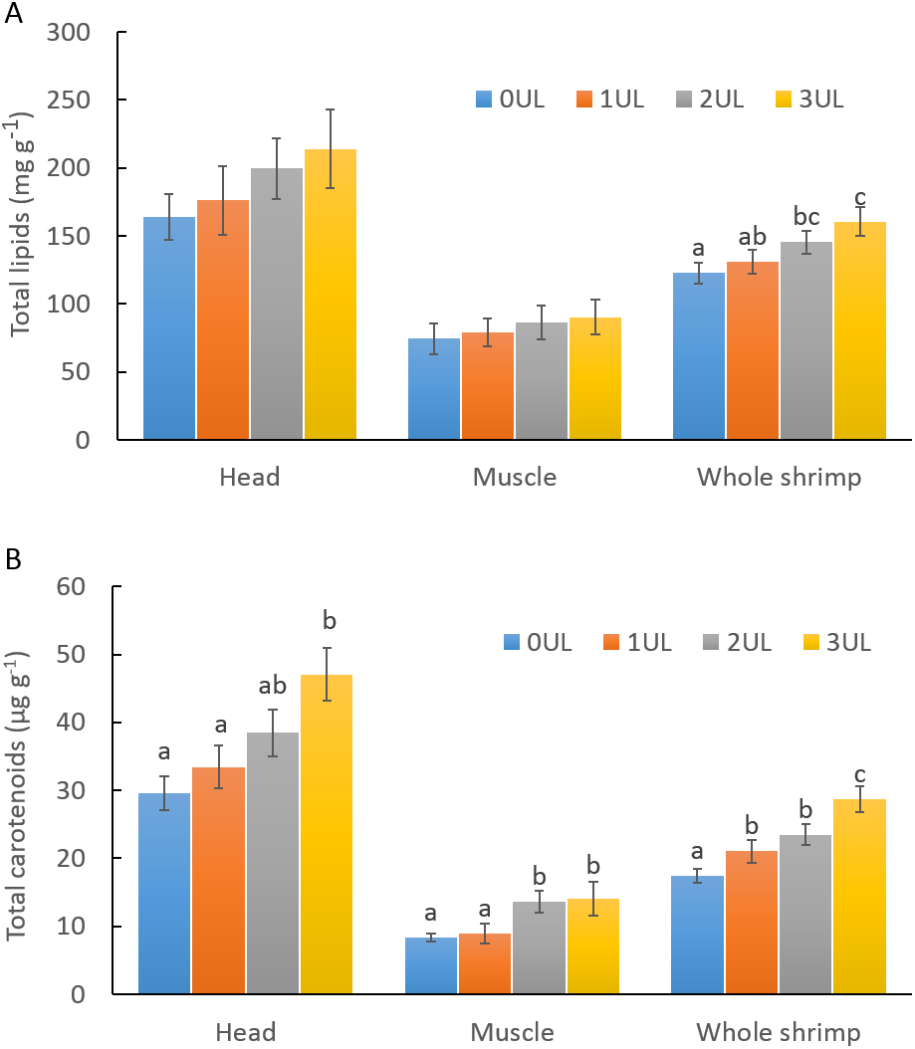
Total lipids and total carotenoid in shrimp fed experimental diets containing different inclusion levels of *U. lactuca* meal. (A) Total lipids and (B) Total carotenoids in shrimp fed experimental diets containing 0% (0UL), 1% (1UL), 2% (2UL) and 3% (3UL) of *U. lactuca* meal. Values are given as mean ± SD of multiple determinations (*n* = 5). Different superscripts denotes statistical differences among treatments (*p* < 0.05).

In feeding trial 2, experimental feeds did no show differences in proximal composition except for ash content in feed containing 3% of *U. lactuca* meal (3UL), which resulted 1.1% higher compared to the control feed (0UL) as expected. Total carotenoids in *U. lactuca* meal resulted in 3.5 mg g^−1^ in dry basis. Results of shrimp performance after evaluation of experimental feeds with different inclusion levels of *U. lactuca* meal (Table 3) showed that shrimp fed diet with 3% *U. lactuca* meal had a significantly higher growth in terms of final weight, weight gain, and SGR (*p* < 0.05) compared to the control diet (0UL) and that of 1% of *U. lactuca* (1UL). Shrimp fed 2UL treatment showed no significant differences in growth parameters compared to the other treatments. In terms of feed utilization, the 3UL diet induced a significantly lower (*p* < 0.05) FCR compared to the rest of the treatments. Shrimp survival was higher than 95% in all treatments. Total lipid content in whole shrimp (Fig. 2A) was significantly higher in shrimp with 3UL compared to the rest of treatments (*p* < 0.05). Additionally, shrimp fed 3UL showed significantly higher concentration of total carotenoids in the head; in the muscle, 2UL and 3UL yielded significantly higher amounts of carotenoids than the rest of treatments; and considering the whole shrimp, all *U. lactuca* meal diets resulted in significantly higher content of carotenoids compared to the control diet (Fig. 2B). Lineal regression model showed a correlation between level of *U. lactuca* meal inclusion in feed and total carotenoids in whole shrimp (*y* = 3.605*x* + 17.22, *R*^2^ = 0.88) and head (*y* = 5.738*x* + 28.518, *R*^2^ = 0.788). Water quality parameters during the second experimental period were very stable among treatments: temperature (28 ± 0.4 °C) pH (8.0 ± 0.1), NH3, NH4+ (<0.5 mg L^−1^), NO_2_ (<0.25 mg L^−1^), and NO_3_ (<5 mg L^−1^).

**Table 3:** Growth performance, feed utilization, and survival after 4-week experimental trial with *L. vannamei* juveniles fed diets containing different levels of *U. lactuca* meal.

	0UL	1UL	2UL	3UL	*P* value
Final weight (g)	2.54 ± 0.08^a^	2.55 ± 0.08^a^	2.58 ± 0.11^a,b^	2.78 ± 0.06^b^	0.026
Weight gain (%)	330 ± 13^a^	332 ± 13^a^	337 ± 19^a,b^	371 ± 10^b^	0.028
SGR (% day^−1^)	5.21 ± 0.11^a^	5.23 ± 0.11^b^	5.27 ± 0.15^a,b^	5.54 ± 0.08^b^	0.030
FC	2.47 ± 0.06	2.44 ± 0.04	2.51 ± 0.06	2.53 ± 0.03	0.163
FCR	1.27 ± 0.03^b^	1.25 ± 0.05^b^	1.26 ± 0.05^b^	1.15 ± 0.03^a^	0.028
Survival (%)	100	96 ± 6	100	100	0.441

**Notes.**

Values are given as mean ± SD of triplicate determinations. Means with different superscripts in same row are significantly different (*p* < 0.05).

## Discussion

According to the water quality parameters during experiment 1, results revealed the high efficiency of *U. lactuca* in removing nitrogen compounds and phosphorus from shrimp’s wastewater (80% and 64%, respectively) under the integrated recirculation system. These results are consistent with other reports describing the high efficiency of *Ulvales* in biofiltering inorganic compounds from aquaculture effluents. Copertino, Tormena & Seeliger (2009) determined that *U. clathrata* removes up to 70–82% of the total ammonia nitrogen (TAN) and 50% of phosphate. In a study of an intensive co-culture system of *U. lactuca* and *L. vannamei*, TAN and phosphate were significantly reduced in culture water by 25.9% and 24.6%, respectively, compared to a system without *U. lactuca* (Brito et al., 2014). The nitrogenous compounds removed by seaweed reflected 15.5% protein content in meal, which revealed a higher proportion than reports in wild collected *U. lactuca* (7.1 to 10.7%) (Wong & Cheung, 2000; Yaich et al., 2011; Tabarsa et al., 2012), but lower than described for *U. lactuca* cultured in a controlled system (21.1%) (Ventura & Castañón, 1998). An integration of a total or partial recirculating system of *U. lactuca* and shrimp may decrease the need of out coming water, improving farm biosecurity and reducing the possibility of disease outbreaks (Muniesa et al., 2015). According to the present experimental results, *U. lactuca* meets different criteria suggested by other authors to select an efficient seaweed biofilter for integrated aquaculture, which includes nutrient intake from wastewater (Kang, Park & Chung, 2011), seaweed density, and water flow rate (Al-Hafedh, Alam & Buschmann, 2015).

Water bioremediation with *U. lactuca* (trial 1) did not affect shrimp growth or feed utilization, as described by Fourooghifard et al. (2017), where the water quality improved without affecting shrimp growth in a zero water exchange system of integrated culture of *L. vannamei* and *Gracilaria cortica.* No significant growth differences were observed in *L.  vannamei* cultured in floating cages with red seaweed *Kappaphycus alvarezii* compared to shrimp monoculture system (Lombardi et al., 2006). On the other hand, when no water exchange was performed, shrimp growth and feed utilization was affected possibly by water quality. It has been described that exposure to high concentrations of ammonia in water increases oxygen and energy demand in shrimp (Racotta & Hernández-Herrera, 2000) reflected in lower growth (Chen & Kou, 1992). However, shrimp performance in low or no water exchange culture systems can also be affected by the shrimp stock densities (Hopkins et al., 1993), feed composition (Wasielesky et al., 2006), and feeding frequency (Tacon et al., 2002).

In feeding trial 2, where *U. lactuca* produced by bioremediation was evaluated as feed ingredient, shrimp growth was improved when fed 3% *U. lactuca* meal in feed. Rodríguez-González et al. (2014) suggest that the limiting inclusion level for *U. lactuca* meal in shrimp feed should not exceed 5%, showing that levels of 10 and 15% reduced significantly shrimp growth compared to a control diet without *U. lactuca* inclusion. Serrano Jr, Santizo & Tumbokon (2015) also experimented with 15 and 30% *U. lactuca* meal inclusion in *P. monodon* shrimp, finding no growth improvement at the lower inclusion level and significant reduction of shrimp growth at the higher inclusion level. Similar results in fish were observed, poor growth and feed utilization were shown in African catfish (Abdel-warith, Younis & Al-asgah, 2016) and in Rainbow trout (Yildirim et al., 2009) when high levels of *U. lactuca* meal (>20% and 10% respectively) were included in feed compared with 0% inclusion, suggesting lower inclusion levels.

Shrimp growth improvement at low inclusion levels were found with other seaweed meals, as for example with 2 or 4% of *Macrocystis pyrifera* (Cruz-Suárez et al., 2000) or *Sargassum sp.* (Suárez-García, 2006) included in shrimp feed. Yu et al. (2016) also recommends low inclusion levels (2 or 3%) of *Gracilaria lemaneiformis* meal in order to improve weight gain in *L. vannamei*. The growth promotor effect, as in the present work, is generally attributed to vitamins, minerals and lipids present in the seaweed (Cruz-suárez et al., 2008; Tabarsa et al., 2012).

*U. lactuca* showed high content of ash (36.5%) similar to the value reported by Rodríguez-González et al. (2014) (41.7%), which could explain the limiting inclusion level of seaweed meal in the feed. High inclusion levels of seaweed meal in feed reflects higher contents of ash, which has been related with decrement of feed digestibility (Brunson, Romaire & Reigh, 1997; Yang et al., 2009). In a study in black tiger shrimp *Penaeus monodon*, apparent digestibility of *U. lactuca* meal was significantly lower (71%) than for protein concentrate from *U. lactuca* (99%) (Santizo, Serrano & Corre, 2014). This decrement of feed digestibility on *Ulva* meal diets is not observed when fresh seaweed is used as food, like when shrimp are fed fresh *U. lactuca* (Pallaoro, do Nascimento Vieira & Hayashi, 2016) or *U. clathrata* (Cruz-Suárez et al., 2010), which, in both cases, could be save at around 50% of pelleted feed without negative effects on shrimp growth.

The increase of 30% in whole shrimp lipid content of shrimps fed 3% *U. lactuca* meal diet, respect to control feed, was also described in *L. vannamei* co-cultured with *U. clathrata,* where a combination of pelleted feed and seaweed increased up to 50% total lipid content in shrimp (Cruz-Suárez et al., 2010). This increase in shrimp lipid content could be partially attributed to carotenoids content in the algae. Carotenoid in crustaceans are responsible for pigmentation and as source of provitamin A and as antioxidants (Liñan Cabello, Paniagua-Michel & Hopkins, 2002). Total carotenoids in the *U. lactuca* meal in the present study was in the range described for the same species and others *Ulvales* (240 to 500 ug g^−1^ fresh weight) (Xia, Li & Zou, 2004; Kumar et al., 2010; Peña Rodríguez et al., 2011). Shrimp fed diets with *U. lactuca* meal significantly increased whole shrimp carotenoid content, with the highest concentration in the head. Penaeid shrimp effectively use carotenoids from *Ulvales* to increase body pigmentation. Shrimp fed fresh *U. clathrata* increase carotenoid content as the use of pelleted food decreased (Cruz-Suárez et al., 2010). In another study, feeds with 3.3% of seaweed (*U. clathrata*) meal inclusion diet resulted in higher shrimp pigmentation after cooking respect to *Ascophilllum nodosum* and *Macrocystis pyrifera* diets (Cruz-Suárez et al., 2009). A diet containing 5% of *Enteromorpha intestinalis* meal increased significantly the astaxanthin content in *P. monodon* muscle compared to a control diet after 30 days of feeding trial (Subhra Bikash, 2015).

## Conclusions

In conclusion, the results of the present study demonstrated that *U. lactuca* can be used on integrated aquaculture systems in terms of nitrogen and phosphate water bioremediation, and the inclusion of the seaweed biomass produced as feed additive improves shrimp growth performance and shrimp carotenoid content.

##  Supplemental Information

10.7717/peerj.4459/supp-1 Supplemental Information 1 Zootechnical parameters on shrimp experimental trials, water quality and total lipid and carotenoid content in shrimp

10.7717/peerj.4459/supp-2 Supplemental Information 2 Lineal regression models between U. lactuca inclusion level in feed and evaluated shrimp parameters Each regression model consider the respective replicates of the evaluated parameters.

10.7717/peerj.4459/supp-3 Supplemental Information 3 Statistics results Includes homogeneity and normality test of the data, ANOVA and Tukey’s test.

## Additional Information and Declarations

### Field Study Permissions

The following information was supplied relating to field study approvals (i.e., approving body and any reference numbers): Wild collection of native Ulva sp. (sea lettuce) was approved by the Secretary of Agriculture, Animal breeding, Rural development, Fisheries and Food (Secretaria de Agricultura, Ganadería, Desarrollo Rural, Pesca y Alimentación) CONAPESCA, México (#PRMN/DGOPA-019/2015).

### Data Availability

The following information was supplied regarding data availability: The raw data generated from experimental trials and chemical analysis are provided as a Supplemental File.
