# Supplementary material for: Use of seaweed Ulva lactuca for water bioremediation and as feed additive for white shrimp Litopenaeus vannamei
Source: PeerJ. 2018 Mar 5;6:e4459. doi: 10.7717/peerj.4459 (PMC5842761; doi:10.7717/peerj.4459)
Supplement: Supplemental Information 2 — Each regression model consider the respective replicates of the evaluated parameters. [file peerj-06-4459-s002.pdf]

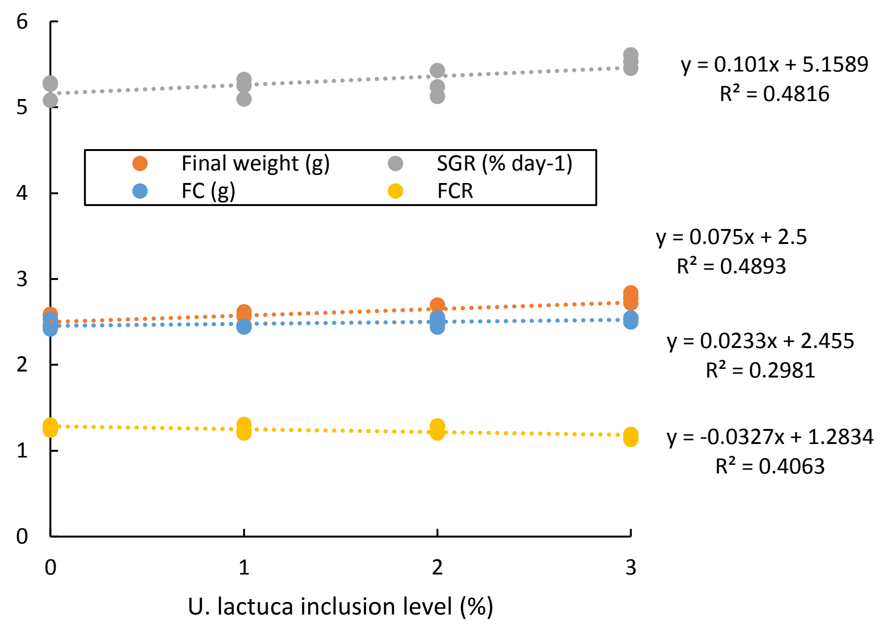

Figure A. Linear regression model between level of U. lactuca meal inclusion in feed and final weight, specific growth rate (SGR), feed consumption (FC) and feed conversion ratio (FCR) of shrimp.

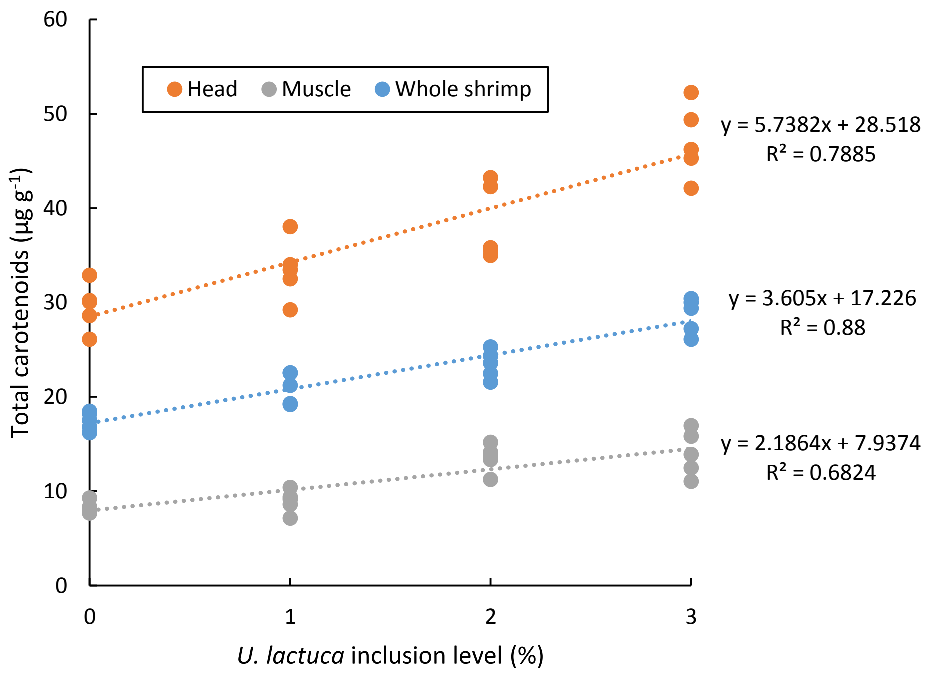

Figure B. Linear regression model between level of U. lactuca meal inclusion in feed and total carotenoids in shrimp.

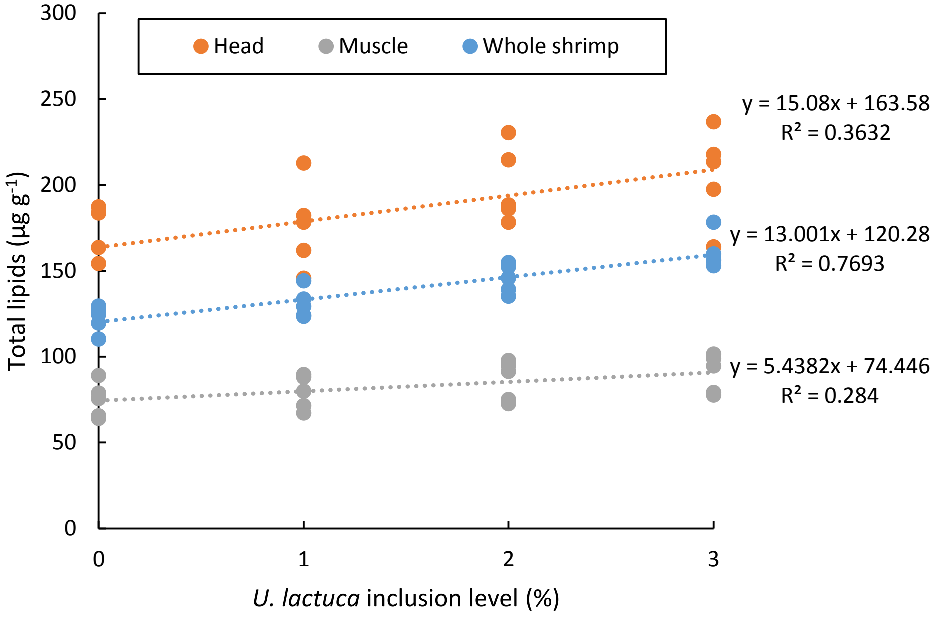

Figure C. Linear regression model between level of U. lactuca meal inclusion in feed and total lipids in shrimp.
