# Supplementary material for: Use of seaweed Ulva lactuca for water bioremediation and as feed additive for white shrimp Litopenaeus vannamei
Source: PeerJ. 2018 Mar 5;6:e4459. doi: 10.7717/peerj.4459 (PMC5842761; doi:10.7717/peerj.4459)
Supplement: Supplemental Information 3 — Includes homogeneity and normality test of the data, ANOVA and Tukey’s test. [file peerj-06-4459-s003.pdf]

Statistical analysis of data

Feeding trial 1

Normality test

	Treatment	Kolmogorov-Smirnov[a]			Shapiro-Wilk		
		Statistic	gl	Sig.	Statistic	gl	Sig.
Finalweight	CWE	.188	3	.	.998	3	.910
	SWE	.284	3	.	.933	3	.500
	NWE	.349	3	.	.832	3	.194
Weightgain	CWE	.188	3	.	.998	3	.910
	SWE	.284	3	.	.933	3	.500
	NWE	.340	3	.	.848	3	.235
SGR	CWE	.185	3	.	.998	3	.924
	SWE	.286	3	.	.931	3	.493
	NWE	.349	3	.	.832	3	.194
FC	CWE	.229	3	.	.981	3	.739
	SWE	.219	3	.	.987	3	.780
	NWE	.219	3	.	.987	3	.780
FCR	CWE	.192	3	.	.997	3	.895
	SWE	.330	3	.	.867	3	.287
	NWE	.191	3	.	.997	3	.900
Survival	CWE	.175	3	.	1.000	3	1.000
	SWE	.385	3	.	.750	3	.000
	NWE	.385	3	.	.750	3	.000

a. Corrección de significación de Lilliefors

Test of homogeneity of variance

	Levene test	gl1	gl2	Sig.
Finalweight	.267	2	6	.774
Weightgain	.260	2	6	.779
SGR	.430	2	6	.669
FC	1.750	2	6	.252
FCR	2.398	2	6	.172
Survival	.364	2	6	.709

Descriptivos

		N	Mean	Standard Deviation	Standard Error	95% confidence interval for the mean		Mínimum	Maximum
						Lower limit	Upper limit		
Finalweight	CWE	3	2.1464	.05706	.03295	2.0047	2.2882	2.09	2.21
	SWE	3	2.0854	.03624	.02092	1.9954	2.1754	2.05	2.12
	NWE	3	1.8167	.04933	.02848	1.6941	1.9392	1.76	1.85
	Total	9	2.0162	.15760	.05253	1.8950	2.1373	1.76	2.21
Weightgain	CWE	3	613.10	18.958	10.945	566.01	660.20	595	633
	SWE	3	592.83	12.038	6.950	562.92	622.73	579	603
	NWE	3	503.67	16.289	9.404	463.20	544.13	485	515
	Total	9	569.87	52.294	17.431	529.67	610.06	485	633
SGR	CWE	3	7.0151	.09487	.05477	6.7794	7.2507	6.92	7.11
	SWE	3	6.9125	.06228	.03596	6.7578	7.0673	6.84	6.96
	NWE	3	6.4233	.09866	.05696	6.1783	6.6684	6.31	6.49
	Total	9	6.7837	.28400	.09467	6.5654	7.0020	6.31	7.11
FC	CWE	3	2.2009	.07755	.04478	2.0083	2.3936	2.12	2.27
	SWE	3	2.1127	.02013	.01162	2.0627	2.1627	2.09	2.13
	NWE	3	2.1433	.05033	.02906	2.0183	2.2684	2.09	2.19
	Total	9	2.1523	.06119	.02040	2.1053	2.1993	2.09	2.27
FCR	CWE	3	1.1925	.00902	.00521	1.1701	1.2149	1.18	1.20
	SWE	3	1.1854	.02141	.01236	1.1322	1.2386	1.17	1.21
	NWE	3	1.4133	.05508	.03180	1.2765	1.5501	1.36	1.47
	Total	9	1.2638	.11613	.03871	1.1745	1.3530	1.17	1.47
Survival	CWE	3	90.00	10.000	5.774	65.16	114.84	80	100
	SWE	3	96.67	5.774	3.333	82.32	111.01	90	100
	NWE	3	83.33	5.774	3.333	68.99	97.68	80	90
	Total	9	90.00	8.660	2.887	83.34	96.66	80	100

ANOVA

		Sum of squares	gl	Half quadratic	F	Sig.
Finalweight	Entre grupos	.185	2	.092	39.565	.000
	Dentro de grupos	.014	6	.002		
	Total	.199	8			
Weightgain	Entre grupos	20337.711	2	10168.856	39.636	.000
	Dentro de grupos	1539.327	6	256.554		

	Total	21877.038	8			
SGR	Entre grupos	.600	2	.300	39.801	.000
	Dentro de grupos	.045	6	.008		
	Total	.645	8			
FC	Entre grupos	.012	2	.006	2.018	.214
	Dentro de grupos	.018	6	.003		
	Total	.030	8			
FCR	Entre grupos	.101	2	.050	42.296	.000
	Dentro de grupos	.007	6	.001		
	Total	.108	8			
Survival	Entre grupos	266.667	2	133.333	2.400	.171
	Dentro de grupos	333.333	6	55.556		
	Total	600.000	8			

Multiple comparison

HSD Tukey

Dependent variable	(I) Treatment	(J) Treatment	Diferencia de medias (I-J)	Standard Error	Sig.	95% confidence interval	
						Lower Limit	Upper Limit
Finalweight	CWE	SWE	.06103	.03945	.336	-.0600	.1821
		NWE	.32978*	.03945	.000	.2087	.4508
	SWE	CWE	-.06103	.03945	.336	-.1821	.0600
		NWE	.26875*	.03945	.001	.1477	.3898
	NWE	CWE	-.32978*	.03945	.000	-.4508	-.2087
		SWE	-.26875*	.03945	.001	-.3898	-.1477
Weightgain	CWE	SWE	20.275	13.078	.335	-19.85	60.40
		NWE	109.438*	13.078	.000	69.31	149.56
	SWE	CWE	-20.275	13.078	.335	-60.40	19.85
		NWE	89.163*	13.078	.001	49.04	129.29
	NWE	CWE	-109.438*	13.078	.000	-149.56	-69.31
		SWE	-89.163*	13.078	.001	-129.29	-49.04
SGR	CWE	SWE	.10254	.07089	.378	-.1150	.3200
		NWE	.59175*	.07089	.000	.3742	.8092
	SWE	CWE	-.10254	.07089	.378	-.3200	.1150
		NWE	.48921*	.07089	.001	.2717	.7067
	NWE	CWE	-.59175*	.07089	.000	-.8092	-.3742

		SWE	-.48921*	.07089	.001	-.7067	-.2717
FC	CWE	SWE	.08826	.04461	.198	-.0486	.2251
		NWE	.05759	.04461	.450	-.0793	.1945
	SWE	CWE	-.08826	.04461	.198	-.2251	.0486
		NWE	-.03067	.04461	.779	-.1675	.1062
	NWE	CWE	-.05759	.04461	.450	-.1945	.0793
		SWE	.03067	.04461	.779	-.1062	.1675
FCR	CWE	SWE	.00711	.02818	.966	-.0793	.0936
		NWE	-.22080*	.02818	.001	-.3073	-.1343
	SWE	CWE	-.00711	.02818	.966	-.0936	.0793
		NWE	-.22791*	.02818	.000	-.3144	-.1415
	NWE	CWE	.22080*	.02818	.001	.1343	.3073
		SWE	.22791*	.02818	.000	.1415	.3144
Survival	CWE	SWE	-6.667	6.086	.551	-25.34	12.01
		NWE	6.667	6.086	.551	-12.01	25.34
	SWE	CWE	6.667	6.086	.551	-12.01	25.34
		NWE	13.333	6.086	.151	-5.34	32.01
	NWE	CWE	-6.667	6.086	.551	-25.34	12.01
		SWE	-13.333	6.086	.151	-32.01	5.34

*. La diferencia de medias es significativa en el nivel 0.05.

Finalweight

HSD Tukey[a]

Treatment	N	Subset for alpha = 0.05	
		1	2
NWE	3	1.8167	
SWE	3		2.0854
CWE	3		2.1464
Sig.		1.000	.336

Se visualizan las medias para los grupos en los subconjuntos homogéneos.

a. Utiliza el tamaño de la muestra de la media armónica = 3.000.

Weightgain

HSD Tukey[a]

Treatment	N	Subset for alpha = 0.05	
		1	2
NWE	3	503.67	
SWE	3		592.83
CWE	3		613.10
Sig.		1.000	.335

Se visualizan las medias para los grupos en los subconjuntos homogéneos.

a. Utiliza el tamaño de la muestra de la media armónica = 3.000.

SGR

HSD Tukey[a]

Treatment	N	Subset for alpha = 0.05	
		1	2
NWE	3	6.4233	
SWE	3		6.9125
CWE	3		7.0151
Sig.		1.000	.378

Se visualizan las medias para los grupos en los subconjuntos homogéneos.

a. Utiliza el tamaño de la muestra de la media armónica = 3.000.

FCR

HSD Tukey[a]

Treatment	N	Subset for alpha = 0.05	
		1	2
SWE	3	1.1854	
CWE	3	1.1925	
NWE	3		1.4133
Sig.		.966	1.000

Se visualizan las medias para los grupos en los subconjuntos homogéneos.

a. Utiliza el tamaño de la muestra de la media armónica = 3.000.

Statistical analysis of data from feeding trial 2

Normality test

	Treatment	Kolmogorov-Smirnov[a]			Shapiro-Wilk		
		Statistic	gl	Sig.	Statistic	gl	Sig.
Finalweight	0UL	.362	3	.	.803	3	.122
	1UL	.263	3	.	.955	3	.593
	2UL	.238	3	.	.976	3	.702
	3UL	.175	3	.	1.000	3	1.000
Weightgain	0UL	.358	3	.	.812	3	.144
	1UL	.271	3	.	.948	3	.559
	2UL	.236	3	.	.977	3	.712
	3UL	.175	3	.	1.000	3	1.000
SGR	0UL	.363	3	.	.802	3	.119
	1UL	.266	3	.	.952	3	.579
	2UL	.233	3	.	.979	3	.723
	3UL	.176	3	.	1.000	3	.989
FC	0UL	.191	3	.	.997	3	.900
	1UL	.385	3	.	.750	3	.000
	2UL	.356	3	.	.818	3	.157
	3UL	.314	3	.	.893	3	.363
FCR	0UL	.175	3	.	1.000	3	1.000
	1UL	.314	3	.	.893	3	.363
	2UL	.385	3	.	.750	3	.000
	3UL	.328	3	.	.871	3	.298
Survival	1UL	.385	3	.	.750	3	.000

a. Corrección de significación de Lilliefors

b. Sobrevivencia es constante cuando Tratamiento = 0 UL. Se ha omitido.

c. Sobrevivencia es constante cuando Tratamiento = 2% UL. Se ha omitido.

d. Sobrevivencia es constante cuando Tratamiento = 3% UL. Se ha omitido.

Test of homogeneity of variance

	Levene test	gl1	gl2	Sig.
Finalweight	.533	3	8	.672
Weightgain	.581	3	8	.644
SGR	.601	3	8	.632
FC	2.967	3	8	.097
FCR	.975	3	8	.451
Survival	16.000	3	8	.001

Descriptives

		N	Mean	Standard Deviation	Standard Error	95% confidence interval for the mean		Mínimum	Maximum
						Lower limit	Upper limit		
Finalweight	0UL	3	2.5400	.07810	.04509	2.3460	2.7340	2.45	2.59
	1UL	3	2.5500	.08185	.04726	2.3467	2.7533	2.46	2.62
	2UL	3	2.5800	.11136	.06429	2.3034	2.8566	2.48	2.70
	3UL	3	2.7800	.06000	.03464	2.6310	2.9290	2.72	2.84
	Total	12	2.6125	.12520	.03614	2.5330	2.6920	2.45	2.84
Weightgain	0UL	3	330.33	13.317	7.688	297.25	363.41	315	339
	1UL	3	332.33	13.868	8.007	297.88	366.78	317	344
	2UL	3	337.33	19.218	11.096	289.59	385.07	320	358
	3UL	3	371.00	10.000	5.774	346.16	395.84	361	381
	Total	12	342.75	21.209	6.123	329.27	356.23	315	381
SGR	0UL	3	5.2124	.11084	.06399	4.9371	5.4878	5.08	5.28
	1UL	3	5.2264	.11529	.06657	4.9400	5.5128	5.10	5.32
	2UL	3	5.2672	.15336	.08854	4.8862	5.6481	5.13	5.43
	3UL	3	5.5355	.07710	.04451	5.3439	5.7270	5.46	5.61
	Total	12	5.3104	.16993	.04905	5.2024	5.4183	5.08	5.61
FC	0UL	3	2.4733	.05508	.03180	2.3365	2.6101	2.42	2.53
	1UL	3	2.4467	.00577	.00333	2.4323	2.4610	2.44	2.45
	2UL	3	2.5100	.06083	.03512	2.3589	2.6611	2.44	2.55
	3UL	3	2.5300	.02646	.01528	2.4643	2.5957	2.50	2.55
	Total	12	2.4900	.04991	.01441	2.4583	2.5217	2.42	2.55
FCR	0UL	3	1.2700	.03000	.01732	1.1955	1.3445	1.24	1.30
	1UL	3	1.2500	.05292	.03055	1.1186	1.3814	1.21	1.31
	2UL	3	1.2633	.04619	.02667	1.1486	1.3781	1.21	1.29

	3UL	3	1.1533	.03215	.01856	1.0735	1.2332	1.13	1.19
	Total	12	1.2342	.06067	.01751	1.1956	1.2727	1.13	1.31
Survival	0UL	3	10.00	.000	.000	10.00	10.00	10	10
	1UL	3	9.67	.577	.333	8.23	11.10	9	10
	2UL	3	10.00	.000	.000	10.00	10.00	10	10
	3UL	3	10.00	.000	.000	10.00	10.00	10	10
	Total	12	9.92	.289	.083	9.73	10.10	9	10

ANOVA

		Sum of squares	gl	Half quadratic	F	Sig.
Finalweight	Entre grupos	.115	3	.038	5.316	.026
	Dentro de grupos	.058	8	.007		
	Total	.172	11			
Weightgain	Entre grupos	3270.250	3	1090.083	5.197	.028
	Dentro de grupos	1678.000	8	209.750		
	Total	4948.250	11			
SGR	Entre grupos	.208	3	.069	5.028	.030
	Dentro de grupos	.110	8	.014		
	Total	.318	11			
FC	Entre grupos	.012	3	.004	2.226	.163
	Dentro de grupos	.015	8	.002		
	Total	.027	11			
FCR	Entre grupos	.027	3	.009	5.196	.028
	Dentro de grupos	.014	8	.002		
	Total	.040	11			
Survival	Entre grupos	.250	3	.083	1.000	.441
	Dentro de grupos	.667	8	.083		
	Total	.917	11			

Multiple comparison

HSD Tukey

			Sig.	95% confidence interval

Dependent variable	(I) Treatment	(J) Treatment	Difference of means (I-J)	Standard Error		Lower Limit	Upper Limit
Finalweight	0UL	1UL	-.01000	.06928	.999	-.2319	.2119
		2UL	-.04000	.06928	.936	-.2619	.1819
		3UL	-.24000*	.06928	.035	-.4619	-.0181
	1UL	0UL	.01000	.06928	.999	-.2119	.2319
		2UL	-.03000	.06928	.971	-.2519	.1919
		3UL	-.23000*	.06928	.042	-.4519	-.0081
	2UL	0UL	.04000	.06928	.936	-.1819	.2619
		1UL	.03000	.06928	.971	-.1919	.2519
		3UL	-.20000	.06928	.078	-.4219	.0219
	3UL	0UL	.24000*	.06928	.035	.0181	.4619
		1UL	.23000*	.06928	.042	.0081	.4519
		2UL	.20000	.06928	.078	-.0219	.4219
Weightgain	0UL	1UL	-2.000	11.825	.998	-39.87	35.87
		2UL	-7.000	11.825	.932	-44.87	30.87
		3UL	-40.667*	11.825	.036	-78.53	-2.80
	1UL	0UL	2.000	11.825	.998	-35.87	39.87
		2UL	-5.000	11.825	.973	-42.87	32.87
		3UL	-38.667*	11.825	.045	-76.53	-.80
	2UL	0UL	7.000	11.825	.932	-30.87	44.87
		1UL	5.000	11.825	.973	-32.87	42.87
		3UL	-33.667	11.825	.083	-71.53	4.20
	3UL	0UL	40.667*	11.825	.036	2.80	78.53
		1UL	38.667*	11.825	.045	.80	76.53
		2UL	33.667	11.825	.083	-4.20	71.53
SGR	0UL	1UL	-.01393	.09578	.999	-.3206	.2928
		2UL	-.05473	.09578	.938	-.3614	.2520
		3UL	-.32303*	.09578	.039	-.6297	-.0163
	1UL	0UL	.01393	.09578	.999	-.2928	.3206
		2UL	-.04080	.09578	.972	-.3475	.2659
		3UL	-.30910*	.09578	.048	-.6158	-.0024
	2UL	0UL	.05473	.09578	.938	-.2520	.3614
		1UL	.04080	.09578	.972	-.2659	.3475
		3UL	-.26830	.09578	.088	-.5750	.0384
	3UL	0UL	.32303*	.09578	.039	.0163	.6297

		1UL	.30910*	.09578	.048	.0024	.6158
		2UL	.26830	.09578	.088	-.0384	.5750
FC	0UL	1UL	.02667	.03528	.872	-.0863	.1396
		2UL	-.03667	.03528	.733	-.1496	.0763
		3UL	-.05667	.03528	.427	-.1696	.0563
	1UL	0UL	-.02667	.03528	.872	-.1396	.0863
		2UL	-.06333	.03528	.342	-.1763	.0496
		3UL	-.08333	.03528	.163	-.1963	.0296
	2UL	0UL	.03667	.03528	.733	-.0763	.1496
		1UL	.06333	.03528	.342	-.0496	.1763
		3UL	-.02000	.03528	.939	-.1330	.0930
	3UL	0UL	.05667	.03528	.427	-.0563	.1696
		1UL	.08333	.03528	.163	-.0296	.1963
		2UL	.02000	.03528	.939	-.0930	.1330
FCR	0UL	1UL	.02000	.03383	.932	-.0883	.1283
		2UL	.00667	.03383	.997	-.1017	.1150
		3UL	.11667*	.03383	.035	.0083	.2250
	1UL	0UL	-.02000	.03383	.932	-.1283	.0883
		2UL	-.01333	.03383	.978	-.1217	.0950
		3UL	.09667	.03383	.081	-.0117	.2050
	2UL	0UL	-.00667	.03383	.997	-.1150	.1017
		1UL	.01333	.03383	.978	-.0950	.1217
		3UL	.11000*	.03383	.047	.0017	.2183
	3UL	0UL	-.11667*	.03383	.035	-.2250	-.0083
		1UL	-.09667	.03383	.081	-.2050	.0117
		2UL	-.11000*	.03383	.047	-.2183	-.0017
Survival	0UL	1UL	.333	.236	.525	-.42	1.09
		2UL	.000	.236	1.000	-.75	.75
		3UL	.000	.236	1.000	-.75	.75
	1UL	0UL	-.333	.236	.525	-1.09	.42
		2UL	-.333	.236	.525	-1.09	.42
		3UL	-.333	.236	.525	-1.09	.42
	2UL	0UL	.000	.236	1.000	-.75	.75
		1UL	.333	.236	.525	-.42	1.09
		3UL	.000	.236	1.000	-.75	.75
	3UL	0UL	.000	.236	1.000	-.75	.75
		1UL	.333	.236	.525	-.42	1.09

	2UL	.000	.236	1.000	-.75	.75

*. La diferencia de medias es significativa en el nivel 0.05.

Finalweight

HSD Tukey[a]

Treatment	N	Subset for alpha = 0.05	
		1	2
0UL	3	2.5400	
1UL	3	2.5500	
2UL	3	2.5800	2.5800
3UL	3		2.7800
Sig.		.936	.078

Se visualizan las medias para los grupos en los subconjuntos homogéneos.

a. Utiliza el tamaño de la muestra de la media armónica = 3.000.

Weightgain

HSD Tukey[a]

Treatment	N	Subset for alpha = 0.05	
		1	2
0UL	3	330.33	
1UL	3	332.33	
2UL	3	337.33	337.33
3UL	3		371.00
Sig.		.932	.083

Se visualizan las medias para los grupos en los subconjuntos homogéneos.

a. Utiliza el tamaño de la muestra de la media armónica = 3.000.

SGR

HSD Tukey[a]

Treatment	N	Subset for alpha = 0.05	
		1	2

0UL	3	5.2124	
1UL	3	5.2264	
2UL	3	5.2672	5.2672
3UL	3		5.5355
Sig.		.938	.088

Se visualizan las medias para los grupos en los subconjuntos homogéneos.

a. Utiliza el tamaño de la muestra de la media armónica = 3.000.

Statistical analysis of data of lipids results from experimental trial 2

Normality test

		Kolmogorov-Smirnov[a]			Shapiro-Wilk		
	Treatment	Statistic	gl	Sig.	Statistic	gl	Sig.
CarotHead	1UL	.229	5	.200*	.965	5	.845
	2UL	.338	5	.063	.779	5	.054
	3UL	.184	5	.200*	.984	5	.956
	0UL	.199	5	.200*	.974	5	.901
CarotMuscle	1UL	.189	5	.200*	.969	5	.866
	2UL	.240	5	.200*	.932	5	.613
	3UL	.172	5	.200*	.970	5	.873
	0UL	.272	5	.200*	.900	5	.412
CarotWholebody	1UL	.243	5	.200*	.828	5	.134
	2UL	.144	5	.200*	.984	5	.955
	3UL	.258	5	.200*	.893	5	.371
	0UL	.197	5	.200*	.941	5	.672
LTHead	1UL	.204	5	.200*	.974	5	.903
	2UL	.295	5	.178	.889	5	.354
	3UL	.210	5	.200*	.953	5	.756
	0UL	.201	5	.200*	.934	5	.625
LTMuscle	1UL	.214	5	.200*	.913	5	.489
	2UL	.269	5	.200*	.844	5	.176
	3UL	.249	5	.200*	.847	5	.184
	0UL	.210	5	.200*	.935	5	.630
LTWholebody	1UL	.191	5	.200*	.894	5	.380

2UL	.193	5	.200*	.940	5	.663
3UL	.334	5	.070	.766	5	.042
0UL	.224	5	.200*	.918	5	.518

*. Esto es un límite inferior de la significación verdadera.

a. Corrección de significación de Lilliefors

Test of homogeneity of variance

	Levene test	gl1	gl2	Sig.
CarotHead	1.128	3	16	.367
CarotMuscle	2.664	3	16	.083
CarotWholebody	1.560	3	16	.238
LTHead	.038	3	16	.990
LTMuscle	.379	3	16	.769
LTWholebody	.055	3	16	.982

Descriptive

						95% confidence interval for the mean			
		N	Mean	Standard Deviation	Standard Error	Lower Limit	Upper Limit	Mínimum	Maximum
CarotHead	1UL	5	33.464	3.1631	1.4146	29.537	37.391	29.3	38.1
	2UL	5	38.388	4.0302	1.8023	33.384	43.392	35.0	43.3
	3UL	5	47.068	3.8907	1.7400	42.237	51.899	42.1	52.3
	0UL	5	29.582	2.4852	1.1114	26.496	32.668	26.1	32.9
	Total	20	37.126	7.4128	1.6575	33.656	40.595	26.1	52.3
CarotMuscle	1UL	5	8.938	1.1998	.5366	7.448	10.428	7.1	10.4
	2UL	5	13.570	1.4628	.6542	11.754	15.386	11.3	15.2
	3UL	5	14.052	2.4110	1.0782	11.058	17.046	11.1	17.0
	0UL	5	8.308	.5931	.2652	7.572	9.044	7.7	9.3
	Total	20	11.217	3.0360	.6789	9.796	12.638	7.1	17.0
CarotWholebody	1UL	5	20.970	1.6618	.7432	18.907	23.033	19.2	22.6
	2UL	5	23.468	1.4792	.6615	21.631	25.305	21.6	25.3
	3UL	5	28.640	1.8697	.8362	26.318	30.962	26.1	30.4
	0UL	5	17.456	.9569	.4279	16.268	18.644	16.2	18.5

	Total	20	22.633	4.4080	.9857	20.570	24.697	16.2	30.4
LTHead	1UL	5	176.072	25.0597	11.2070	144.956	207.188	145.6	212.6
	2UL	5	199.468	22.0164	9.8460	172.131	226.805	178.2	230.4
	3UL	5	205.866	27.3863	12.2475	171.861	239.871	163.8	236.8
	0UL	5	163.398	23.8506	10.6663	133.784	193.012	128.5	187.2
	Total	20	186.201	28.7024	6.4181	172.768	199.634	128.5	236.8
LTMuscle	1UL	5	79.224	9.8230	4.3930	67.027	91.421	67.3	89.5
	2UL	5	86.344	11.6869	5.2265	71.833	100.855	72.7	97.7
	3UL	5	90.300	11.2248	5.0199	76.363	104.237	77.6	101.5
	0UL	5	74.546	10.2337	4.5766	61.839	87.253	63.9	89.0
	Total	20	82.604	11.7048	2.6173	77.125	88.082	63.9	101.5
LTWholebody	1UL	5	130.874	8.5140	3.8076	120.302	141.446	123.4	144.2
	2UL	5	145.466	8.3489	3.7337	135.100	155.832	135.3	154.8
	3UL	5	160.640	10.1094	4.5210	148.088	173.192	153.0	178.2
	0UL	5	122.166	7.5855	3.3924	112.747	131.585	110.3	129.4
	Total	20	139.787	17.0030	3.8020	131.829	147.744	110.3	178.2

ANOVA

		Sum of squares	gl	Half quadratic	F	Sig.
CarotHead	Entre grupos	853.791	3	284.597	23.936	.000
	Dentro de grupos	190.242	16	11.890		
	Total	1044.033	19			
CarotMuscle	Entre grupos	136.150	3	45.383	18.631	.000
	Dentro de grupos	38.975	16	2.436		
	Total	175.125	19			
CarotWholebody	Entre grupos	331.741	3	110.580	47.251	.000
	Dentro de grupos	37.444	16	2.340		
	Total	369.185	19			
LTHead	Entre grupos	5926.495	3	1975.498	3.250	.050
	Dentro de grupos	9726.279	16	607.892		
	Total	15652.774	19			
LTMuscle	Entre grupos	747.859	3	249.286	2.150	.134
	Dentro de grupos	1855.189	16	115.949		
	Total	2603.047	19			
LTWholebody	Entre grupos	4285.199	3	1428.400	18.923	.000

	Dentro de grupos	1207.727	16	75.483		
	Total	5492.926	19			

Multiple comparisons

HSD Tukey

Dependent variable	(I) Treatment	(J) Treatment	Difference of means (I-J)	Standard Error	Sig.	95% confidence interval Lower Limit	Upper Limit
CarotHead	1UL	2UL	-4.9240	2.1808	.150	-11.163	1.315
		3UL	-13.6040*	2.1808	.000	-19.843	-7.365
		0UL	3.8820	2.1808	.318	-2.357	10.121
	2UL	1UL	4.9240	2.1808	.150	-1.315	11.163
		3UL	-8.6800*	2.1808	.005	-14.919	-2.441
		0UL	8.8060*	2.1808	.005	2.567	15.045
	3UL	1UL	13.6040*	2.1808	.000	7.365	19.843
		2UL	8.6800*	2.1808	.005	2.441	14.919
		0UL	17.4860*	2.1808	.000	11.247	23.725
	0UL	1UL	-3.8820	2.1808	.318	-10.121	2.357
		2UL	-8.8060*	2.1808	.005	-15.045	-2.567
		3UL	-17.4860*	2.1808	.000	-23.725	-11.247
CarotMuscle	1UL	2UL	-4.6320*	.9871	.001	-7.456	-1.808
		3UL	-5.1140*	.9871	.000	-7.938	-2.290
		0UL	.6300	.9871	.918	-2.194	3.454
	2UL	1UL	4.6320*	.9871	.001	1.808	7.456
		3UL	-.4820	.9871	.961	-3.306	2.342
		0UL	5.2620*	.9871	.000	2.438	8.086
	3UL	1UL	5.1140*	.9871	.000	2.290	7.938
		2UL	.4820	.9871	.961	-2.342	3.306
		0UL	5.7440*	.9871	.000	2.920	8.568
	0UL	1UL	-.6300	.9871	.918	-3.454	2.194
		2UL	-5.2620*	.9871	.000	-8.086	-2.438
		3UL	-5.7440*	.9871	.000	-8.568	-2.920
CarotWholebody	1UL	2UL	-2.4980	.9675	.084	-5.266	.270
		3UL	-7.6700*	.9675	.000	-10.438	-4.902
		0UL	3.5140*	.9675	.011	.746	6.282
	2UL	1UL	2.4980	.9675	.084	-.270	5.266

		3UL	-5.1720*	.9675	.000	-7.940	-2.404
		0UL	6.0120*	.9675	.000	3.244	8.780
	3UL	1UL	7.6700*	.9675	.000	4.902	10.438
		2UL	5.1720*	.9675	.000	2.404	7.940
		0UL	11.1840*	.9675	.000	8.416	13.952
	0UL	1UL	-3.5140*	.9675	.011	-6.282	-.746
		2UL	-6.0120*	.9675	.000	-8.780	-3.244
		3UL	-11.1840*	.9675	.000	-13.952	-8.416
LTHead	1UL	2UL	-23.3960	15.5935	.460	-68.009	21.217
		3UL	-29.7940	15.5935	.263	-74.407	14.819
		0UL	12.6740	15.5935	.848	-31.939	57.287
	2UL	1UL	23.3960	15.5935	.460	-21.217	68.009
		3UL	-6.3980	15.5935	.976	-51.011	38.215
		0UL	36.0700	15.5935	.136	-8.543	80.683
	3UL	1UL	29.7940	15.5935	.263	-14.819	74.407
		2UL	6.3980	15.5935	.976	-38.215	51.011
		0UL	42.4680	15.5935	.065	-2.145	87.081
	0UL	1UL	-12.6740	15.5935	.848	-57.287	31.939
		2UL	-36.0700	15.5935	.136	-80.683	8.543
		3UL	-42.4680	15.5935	.065	-87.081	2.145
LTMuscle	1UL	2UL	-7.1200	6.8103	.726	-26.604	12.364
		3UL	-11.0760	6.8103	.393	-30.560	8.408
		0UL	4.6780	6.8103	.901	-14.806	24.162
	2UL	1UL	7.1200	6.8103	.726	-12.364	26.604
		3UL	-3.9560	6.8103	.936	-23.440	15.528
		0UL	11.7980	6.8103	.340	-7.686	31.282
	3UL	1UL	11.0760	6.8103	.393	-8.408	30.560
		2UL	3.9560	6.8103	.936	-15.528	23.440
		0UL	15.7540	6.8103	.136	-3.730	35.238
	0UL	1UL	-4.6780	6.8103	.901	-24.162	14.806
		2UL	-11.7980	6.8103	.340	-31.282	7.686
		3UL	-15.7540	6.8103	.136	-35.238	3.730
LTWholebody	1UL	2UL	-14.5920	5.4948	.074	-30.313	1.129
		3UL	-29.7660*	5.4948	.000	-45.487	-14.045
		0UL	8.7080	5.4948	.414	-7.013	24.429
	2UL	1UL	14.5920	5.4948	.074	-1.129	30.313
		3UL	-15.1740	5.4948	.060	-30.895	.547

		0UL	23.3000*	5.4948	.003	7.579	39.021
	3UL	1UL	29.7660*	5.4948	.000	14.045	45.487
		2UL	15.1740	5.4948	.060	-.547	30.895
		0UL	38.4740*	5.4948	.000	22.753	54.195
	0UL	1UL	-8.7080	5.4948	.414	-24.429	7.013
		2UL	-23.3000*	5.4948	.003	-39.021	-7.579
		3UL	-38.4740*	5.4948	.000	-54.195	-22.753

*. La diferencia de medias es significativa en el nivel 0.05.

CarotHead

HSD Tukey[a]

treatment	N	Subset for alpha = 0.05		
		1	2	3
0UL	5	29.582		
1UL	5	33.464	33.464	
2UL	5		38.388	
3UL	5			47.068
Sig.		.318	.150	1.000

Se visualizan las medias para los grupos en los subconjuntos homogéneos.

a. Utiliza el tamaño de la muestra de la media armónica = 5.000.

CarotMuscle

HSD Tukey[a]

treatment	N	Subset for alpha = 0.05	
		1	2
0UL	5	8.308	
1UL	5	8.938	
2UL	5		13.570
3UL	5		14.052
Sig.		.918	.961

Se visualizan las medias para los grupos en los subconjuntos homogéneos.

a. Utiliza el tamaño de la muestra de la media armónica = 5.000.

CarotWholebody

HSD Tukey[a]

treatment	N	Subset for alpha = 0.05		
		1	2	3
0UL	5	17.456		
1UL	5		20.970	
2UL	5		23.468	
3UL	5			28.640
Sig.		1.000	.084	1.000

Se visualizan las medias para los grupos en los subconjuntos homogéneos.

a. Utiliza el tamaño de la muestra de la media armónica = 5.000.

LTWholebody

HSD Tukey[a]

treatment	N	Subset for alpha = 0.05		
		1	2	3
0UL	5	122.166		
1UL	5	130.874	130.874	
2UL	5		145.466	145.466
3UL	5			160.640
Sig.		.414	.074	.060

Se visualizan las medias para los grupos en los subconjuntos homogéneos.

a. Utiliza el tamaño de la muestra de la media armónica = 5.000.
